# Effective Delivery of Anti-Cancer Drug Molecules with Shape Transforming Liquid Metal Particles

**DOI:** 10.3390/cancers11111666

**Published:** 2019-10-27

**Authors:** Dasom Kim, Jangsun Hwang, Yonghyun Choi, Yejin Kwon, Jaehee Jang, Semi Yoon, Jonghoon Choi

**Affiliations:** School of Integrative Engineering, Chung-Ang University, Seoul 06974, Korea; stvg54@gmail.com (D.K.); isnickawesome@gmail.com (J.H.); dydgus5057@gmail.com (Y.C.); angang1027@gmail.com (Y.K.); jjaeh95@gmail.com (J.J.); semi103306@gmail.com (S.Y.)

**Keywords:** liquid metal, EGaIn, photothermal, drug delivery system, vascular embolism, doxorubicin

## Abstract

Liquid metals are being studied intensively because of their potential as a drug delivery system. Eutectic gallium–indium (EGaIn) alloy liquid metals have a low melting point, low toxicity, and excellent tissue permeability. These properties may enable them to be vascular embolic agents that can be deformed by light or heat. In this study, we developed EGaIn particles that can deliver anticancer drugs to tumor cells in vitro and change their shapes in response to external stimuli. These particles were prepared by sonicating a solution containing EGaIn and amphiphilic lipids. The liquid metal (LM)/amphiphilic lipid (DSPC, 1,2-distearoyl-sn-glycero-3-phosphocholin) particles formed a vehicle for doxorubicin, an anticancer drug, which was released (up to 50%) when the shape of the particles was deformed by light or heat treatment. LM/DSPC particles are non-toxic and LM/DSPC/doxorubicin particles have anticancer effects (resulting in a cell viability of less than 50%). LM/DSPC/doxorubicin particles were also able to mimic blood vessel embolisms by modifying their shape using precisely controlled light and heat in engineered microchannels. The purpose of this study was to examine the potential of EGaIn materials to treat tumor tissues that cannot be removed by surgery.

## 1. Introduction

The development of new and effective drug carriers to treat cancer is crucial and ongoing [1]. These carriers are usually comprised of biomaterials and are designed to transport small molecules, proteins, DNA, and RNA [2]. When designing drug carriers, aspects such as biocompatibility and biodegradation are carefully considered [3]. Also, they should have low cytotoxicity, and they should be readily absorbed by cells. Drug carriers using metal particles have been widely studied because they are stable, their surface is easy to modify, they are accessible to various drugs, and they are easy to image with MRI or X-ray scanning [4,5].

Metals that are liquid at room temperature are known for their fluidity and conductivity. Mercury, a well-known room temperature liquid metal, is difficult to use in bioresearch because of its toxicity [6]. Alloys such as liquid metals (LM), gallium, gallium–indium common alloys (EGaIn, 75% Ga, 25% In), and galinstan (a liquid metal alloy composed from a family of eutectic alloys mainly consisting of gallium, indium, and tin) are promising alternatives to mercury because of their relatively low toxicity [7,8]. Gallium-based liquid metals have gained the attention of researchers because they are easily formable, deformable, and stretchable. They are also chemically stable and do not react with water at room temperature [9,10,11]. In contrast to mercury, unique properties such as high surface tension, good mobility, high electrical conductivity, good biocompatibility, and low toxicity make LM an attractive material for biomedical applications, as well as microfluidic systems such as circuits, pumps, electrodes, and sensors [12,13,14,15,16]. In particular, colloids of liquid metals are applied to pumps, sensors, catalysts, and drug delivery systems [17]. LM particles are easily produced by sonication, and can also cause vascular embolisms [18]. They have excellent photochemical and photothermal conversion properties, which can be applied to photothermal conversion agents (PTA) and the photothermal therapy (PTT) of tumors [19,20,21,22]. LM particles, however, possess drawbacks that require further research to address. They may cause embolisms in normal tissues; therefore, they must be administered directly to the target vessel using a catheter or stent. Also, their size can be difficult to control after inducing them to change. Furthermore, because no in vivo experiments were performed in this study, a toxicity assessment could not be conducted, and the potential toxicity of the LM particles was not characterized.

Tumor cell proliferation and differentiation are caused by a variety of growth factors, as well as a rich supply of oxygen and nutrients from blood vessels [23]. As tumor growth depends on blood vessels, starving tumors with vascular target therapy is a promising area of study, and research on angiogenesis inhibitors is ongoing [24,25,26]. However, because tumors become resistant to inhibitors, a new focus of research is on vascular blockage [27]. Several agents that can block blood vessels, including small molecule materials, have been studied along with several physical embolic methods such as coils, balloons, and nanoparticle embolics [28,29].

**Figure 1:**
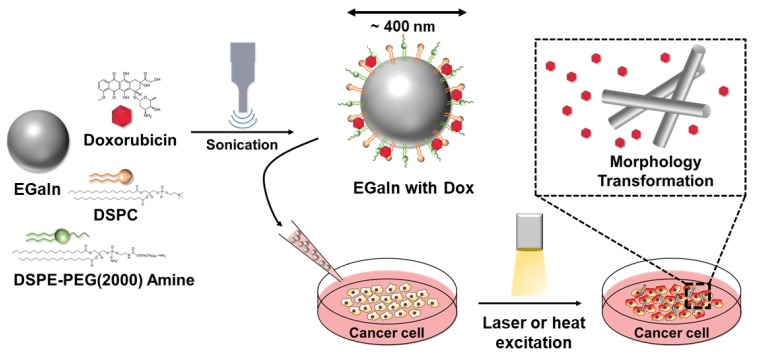
Schematic illustration showing the synthesis of doxorubicin-loaded core-shell liquid metal particles (liquid metal (LM)/1,2-distearoyl-sn-glycero-3-phosphocholin (DSPC)/doxorubicin (DOX)). DSPE-PEG-2000 Amine, 1,2-distearoyl-sn-glycero-3-phosphoethanolamine-N-[amino (polyethylene glycol)-2000]; EGaIn, eutectic gallium–indium.

In this study, engineered liquid metal particles caused in vitro cancer cell necrosis through vascular embolization and conventional drug delivery methods (Figure 1). Using simple sonication techniques, liquid metal particles carrying doxorubicin, an anticancer agent, were formed, and their efficacy was verified. Furthermore, the shape of the liquid metal particles was modified to mimic vascular embolism and verify their potential as a vascular embolizing agent.

## 2. Materials and Methods

### 2.1. Materials

All reagents, unless otherwise specified, were purchased from Sigma-Aldrich (St. Louis, MO, USA). 1,2-distearoyl-sn-glycero-3-phosphoethanolamine-N-[amino (polyethylene glycol)-2000] (DSPE-PEG-2000 Amine) and 1,2-distearoyl-sn-glycero-3-phosphocholin (DSPC), which were used to synthesize the particles, were purchased from Avanti Polar Lipids (Alabaster, AL, USA). MDA-MB-231 breast cancer cells, Hs578T breast cancer cells, and MIA-Paca-2 pancreatic cancer cells were purchased from ATCC (HTB-26, HTB-126, and CRL-1420) (Manassas, VA, USA). A LIVE⁄DEAD^®^ Viability/Cytotoxicity Kit was used to analyze cell viability (Invitrogen, Carlsbad, CA, USA), and a Cell Counting Kit-8 (CCK-8) was obtained from Dojindo (Rockville, MD, USA). A dialysis bag (MW cut-off of 6–8 kDa) was purchased from Spectrumlabs (Piraeus, Greece) to investigate the drug release behavior of the metal particles.

### 2.2. Preparation of the LM/DSPC/Doxorubicin (DOX) Particles

As a general particle synthesis method, we added 10 mg of eutectic gallium–indium (EGaIn) into a 50 mL disposable sample vial and added 80 μL of DSPC (25 mg/mL in chloroform) and 400 μL of DSPE-PEG-2000 Amine (25 mg/mL in chloroform) [7,20,30]. We then placed the vial in a bath sonicator (2501E-DTH, BRANSON) and processed it for 10 min at 50 °C. Next, the chloroform was removed in a dry oven, leaving a powder behind. Then, 10 mL of deionized (DI) water was added to the powder, mixed, and dispersed using probe sonication (VCX750, SONICS) for one hour with an amplitude of 26% and a pulse of 5 s on/5 s pause. The samples were then centrifuged at 15,000× *g* for 10 min, washed twice with 10 mL of DI water, and left for one hour to remove large particles by gravity. To load the doxorubicin, 4 mg of doxorubicin was dissolved in 4 mL of DMSO, 2 μL of triethanolamine (TEA) was added, and the mixture was incubated at room temperature for 12 h. After the reaction, doxorubicin (1 mg/mL) was added to the sonicated sample particles so that the ratio of EGaIn and doxorubicin was 10:1 and rocked at 4 °C for 12 h. After rocking, the samples were centrifuged at 15,000× *g* for 10 min to remove free doxorubicin and rehydrated in 10 mL of DI water or 1× Dulbecco’s phosphate buffered saline (DPBS, pH 7.5). The final product was stored at 4 °C.

### 2.3. Transmission Electron Microscopy (TEM) and Energy Dispersive X-ray Spectroscopy (EDS)

To analyze the shape of the synthesized LM/DSPC/DOX particles, the sample was placed on a copper grid and dried. Then, TEM (Talos L120C, FEI, FEI, Hillsboro, OR, USA) analysis was performed at 120 kV. Negative staining was not performed owing to the clear brightness difference of the metal particles. EDS imaging and mapping of gallium and indium (JEM-F2000, JEOL) were performed to analyze the composition of the particles.

### 2.4. Dynamic Light Scattering (DLS) Analysis

To determine the size of the particles that contained DSPC and DSPE-PEG-2000-Amine in EGaIn, samples were diluted with water at a ratio of 20:1 and analyzed by dynamic light scattering (DLS) (Zetasizer Nano Zs, Malvern, Malvern Panalytical Ltd., Malvern, UK). The samples were measured six times with a reflective index of 3.9 and an absorption intensity of 0.13.

### 2.5. Ultraviolet and Visible Spectroscopy Analysis

The absorption wavelength of the LM particles, particles synthesized with DSPC and DSPE-PEG-2000-Amine, and LM particles loaded with doxorubicin was measured with a UV/vis spectrophotometer (BIOMATE 3S, Thermo, Thermo, Waltham, MA, USA). The samples were measured in a 12.5 × 12.5 × 45 mm cuvette at 300 nm and 900 nm. DI water was used as the blank.

### 2.6. DOX-Loading Efficiency and In Vitro Release Test

To quantitatively analyze the doxorubicin, a standard curve was established using serial dilutions of a stock solution containing 50 μg/mL of doxorubicin in DI water. The fluorescence of each dilution was measured using an excitation wavelength of 480 nm and an emission wavelength of 560 nm.

The drug loading efficiency during the synthesis of the LM/DSPC/DOX particles was confirmed by centrifuging the samples for 10 min at 15,000× *g* and removing the remaining doxorubicin that was not loaded by taking out the supernatant. Using the doxorubicin standard curve as a reference, the fluorescence values of free doxorubicin were analyzed using a multi-plate reader (Synergy H1, BioTek, BioTek, Winooski, VT, USA) at λex 480 nm and λem 560 nm. The drug-loading efficiency was calculated by measuring the free DOX concentrations. In addition, to confirm the release behavior of the drug from the LM/DSPC/DOX particles, 1 mL of the sample was placed into a dialysis bag and stored in 5 mL of 1× DPBS. Then, 1 mL of the diffused drug was sampled at various time points during the incubation (e.g., 1 h, 2 h, 3 h, 6 h, 9 h, 12 h, 24 h, 48 h, and 72 h) at 37 °C to measure the fluorescence, and 1 mL of 1× DPBS was added back to perform an accumulative release.

### 2.7. Confocal Laser Scanning Microscopy

MDA-MB-231 breast cancer cells were used to confirm the behavior, intracellular penetration, and anticancer effects of the LM/DSPC/DOX particles. MDA-MB-231 cells (1.0 × 10^5^ cells/well in an eight-well glass bottom chamber) were incubated in high glucose (Dulbecco’s Modified Eagle Medium) DMEM containing 5% (Fetal Bovine Serum) FBS and 1% penicillin/streptomycin in a 37 °C incubator at 5% CO_2_. After seeding the cells, they were incubated for one day. LM/DSPC/DOX particles were then added at a concentration of 3 μg/mL. The control group was treated with 3 μg/mL of doxorubicin. The samples were incubated for eight hours, washed with 1× DPBS, and fixed with 4% paraformaldehyde. The cells were stained with (4′,6-Diamidine-2′-phenylindole dihydrochloride) DAPI and analyzed using a confocal laser scanning microscope (LSM710, Carl Zeiss, Oberkochen, Germany) at a λ_ex_ of 405 nm (DAPI) and at a λ_ex_ of 488 nm (DOX).

### 2.8. Live/Dead Assays

MDA-MB-231 breast cancer cells were used to determine the cell delivery rate and cell death caused by the LM/DSPC/DOX particles. The cells were seeded at a concentration of 5.0 × 10^3^ cells/well in a 96-well culture plate and incubated at 37 °C with 5% CO_2_ for one day. Subsequently, the LM/DSPC/DOX particles were added to the cells at a low (1 μg/mL) and high (20 μg/mL) concentration and incubated for eight hours. After washing with 1× DPBS, 100 μL of each live/dead assay reagent was added, and the cells were incubated at room temperature for 30 min. After the reagent was removed, the cells were washed again with 1× DPBS and observed using a fluorescence microscope (OX.2053-PLPH, Euromex, Arnhem, The Netherlands).

### 2.9. Cytotoxicity Assays

MDA-MB-231 breast cancer cells, Hs578T breast cancer cells, and MIA-Paca pancreatic cancer cells were used to analyze the time- and concentration-specific cytotoxicity of the LM/DSPC particles. Each cell type was seeded at a concentration of 5 × 10^3^ cells/well in a 96-well culture plate and incubated at 37 °C with 5% CO_2_ for one day. In the hourly cytotoxicity test, MDA-MD-231 cells were treated with control and LM/DSPC/DOX particles using a doxorubicin concentration of 3 μg/mL. After washing with 1× DPBS, the cells were treated with 10% CCK-8 reagent and incubated at 37 °C for two hours. The absorbance was then measured at 450 nm using a multi plate reader.

### 2.10. Light- and Heat-Driven Morphology Changes of LM/DSPC/DOX Particles

The photothermal/thermal characteristics of the LM/DSPC/DOX particles were observed using laser irradiation or 70 °C heat treatment. Laser irradiation used a repetition rate of 100 kHz, pulse width of 200 ns, scan speed of 100 mm/s, loop of 10, and power of 2 W. To conduct the heat treatment, the particles were incubated in a dry oven for 30 min at 50, 60, or 70 °C. Morphological changes were analyzed using TEM according to the experimental conditions outlined above. DLS was used before and after the light/heat treatment to analyze particle sizes according to the particle shape change.

### 2.11. Membrane Blockage Caused by the Shape Transformation of LM/DSPC/DOX Particles

In order to evaluate the blood vessel embolization potential of LM particles, a membrane perfusion experiment using LM/DSPC particles before and after heat treatment was performed. The membrane was a 40 μm pore size nylon cell strainer and the volume of LM/DSPC particles used in this experiment was 1 mL. The absorbance of the particles was measured between 300 nm and 900 nm wavelengths before and after passing through the membrane. After the light and temperature treatment (70 °C, 30 min), the same measurement was performed. The area of the absorbance range was used to analyze the membrane passage rates altered by the shape variations of particles after the light and temperature treatment.

### 2.12. Microfluidic Chip Embolization

Embolization experiments were carried out using a microfluidic chip introduced to LM/DSPC/DOX particles. A (Polydimethylsiloxane) PDMS chip 20 mm in length, 10 μm in depth, and 10 μm in width was fabricated, treated with O_2_ plasma (FEMTO SCIENCE, Gyeonggi-Do, Korea) at 80 W for three minutes, and attached to slide glass. Then, 10 μL of LM/DSPC/DOX particles at a concentration of 5 mg/mL were introduced to the microfluidic chip. They then blocked the channel in the chip after heating it to 70 °C for 30 min. The shape changes and channel blocking behavior of the LM/DSPC/DOX particles were observed using a phase contrast microscope.

## 3. Results and Discussion

### 3.1. The Physiological Properties of LM/DSPC/DOX Particles

**Figure 2:**
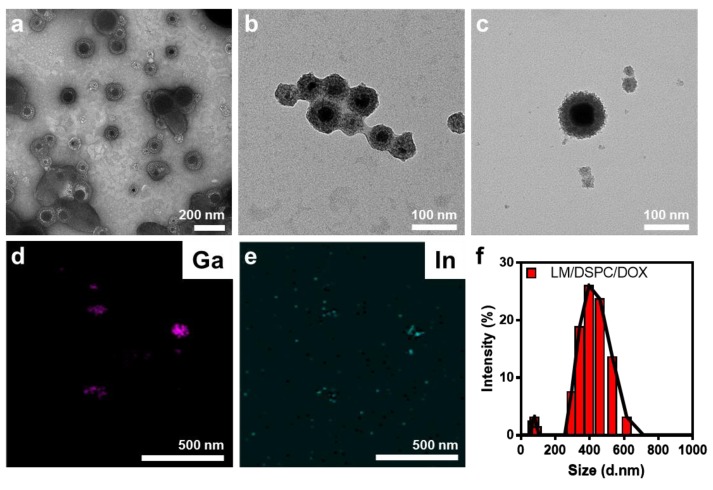

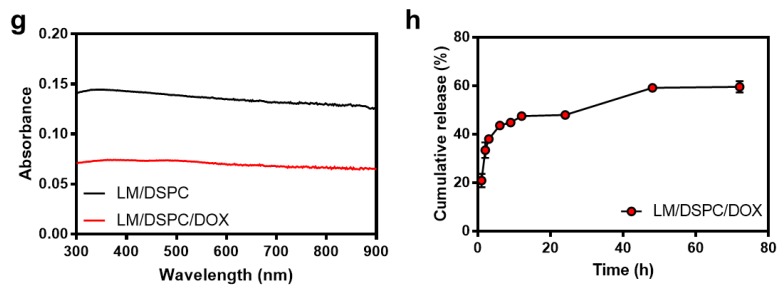
Characteristics of the LM/DSPC/DOX particles. (**a**–**c**) Morphology analysis of the LM/DSPC/DOX particles using transmission electron microscopy (TEM) and (**d**,**e**) energy dispersive X-ray spectroscopy (EDS) mapping. (**f**) Size distribution of the LM/DSPC/DOX particles. (**g**) UV spectroscopy of the LM/DSPC and LM/DSPC/DOX particles. (**h**) Cumulative doxorubicin release of the LM/DSPC/DOX particles.

The LM/DSPC/DOX particles formed a reddish-gray solution after sonication and were analyzed by TEM. They had a core-shell form of lipids with an EGaIn core (Figure 2a–c). Core-shell particles of various sizes were identified (Supplementary Figure S1). The particles consisted of 75% gallium, 20% indium, and 5% oxygen, which maintained their initial content (Figure 2d,e). The average diameter of the particles was 500 nm with a size distribution ranging from tens to hundreds of nanometers (Figure 2f and Supplementary Figure S1). Surface analysis using (Field Emission Scanning Electron Microsope) FE-SEM also showed spherical particles and various size distributions (Supplementary Figure S2). When the absorbance of the synthesized particles was measured, they had a very broad absorbance value in all wavelength ranges measured (from UV to near-infrared regions (NIR)) (Figure 2g). At a wavelength of 808 nm, the mass extinction coefficient of the particles was higher than that of gold nanorods, which are often used as photothermal conversion agents [31]. Therefore, an 808 nm laser was chosen to increase the local temperature and for its biocompatibility in the in vivo light processing experiments. Free doxorubicin not loaded onto the particles was removed to confirm a loading efficiency of approximately 30%. In a drug release test of LM/DSPC/DOX particles loaded with 10 μg/mL of DOX, the drug was released continuously at a rate of up to 60% discharge over 72 h (Figure 2h).

### 3.2. Cellular Uptake of LM/DSPC/DOX

**Figure 3:**
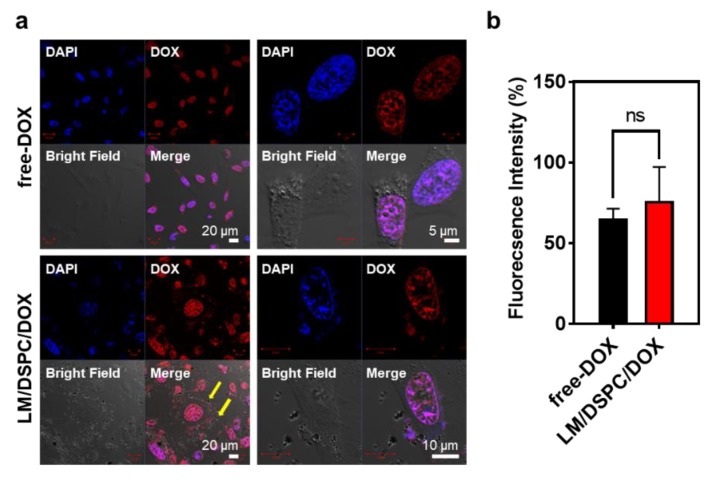
The cellular uptake of doxorubicin (3 μg/mL) and LM/DSPC/DOX particles. (**a**) Confocal microscopy of MDA-MB-231 breast cancer cells after an 8 h incubation with doxorubicin (red) and LM/DSPC/DOX particles (red). The nuclei were stained with (4′,6-Diamidine-2′-phenylindole dihydrochloride) DAPI (blue). (**b**) Fluorescence intensity comparison verifying cellular uptake efficacy (*p* = 0.2748). (ns = no significant).

After LM/DSPC/DOX particles were introduced to MBA-MD-231 cells, it was confirmed that they were taken up by the cells, the drug molecules were released, and the drug was transported into the nucleus. As a result, the fluorescence intensity appeared higher in the experimental group treated with LM/DSPC/DOX particles when compared with the control group treated with the same concentration (3 μg/mL) of free doxorubicin. The drug penetration into the cells was significant (Figure 3a). Comparing the fluorescence intensity of the cells with image J showed that there was no statistically significant difference between the control group and the experimental group (*n* = 10, *p* = 0.2748, Figure 3b). However, the enlarged images in Figure 3a show that the drug was delivered to the nucleus of the cell in the experimental group. Larger aggregated particles remain around the cytoplasm of the cell (yellow arrow), suggesting that the smaller particulates effectively migrated into the cell. The particles can be observed around the cytoplasm of the cell even in cells treated with LM/DSPC particles (Supplementary Figure S3a,b). Furthermore, when LM/DSPC/DOX particles were analyzed using confocal microscopy, doxorubicin fluorescence was observed on the particles themselves or around the particles, indicating that the drug had, in fact, been loaded onto the particles (Supplementary Figure S3c,d). Thus, it was confirmed that the liquid metal nanoparticles were effectively absorbed by the cancer cells and that doxorubicin was effectively transported into the nucleus of cells within a given time. These data show that LM/DSPC/DOX particles can be used as drug carriers.

### 3.3. The Effect of LM/DSPC/DOX Particle Drug Delivery on Cell Death

**Figure 4:**
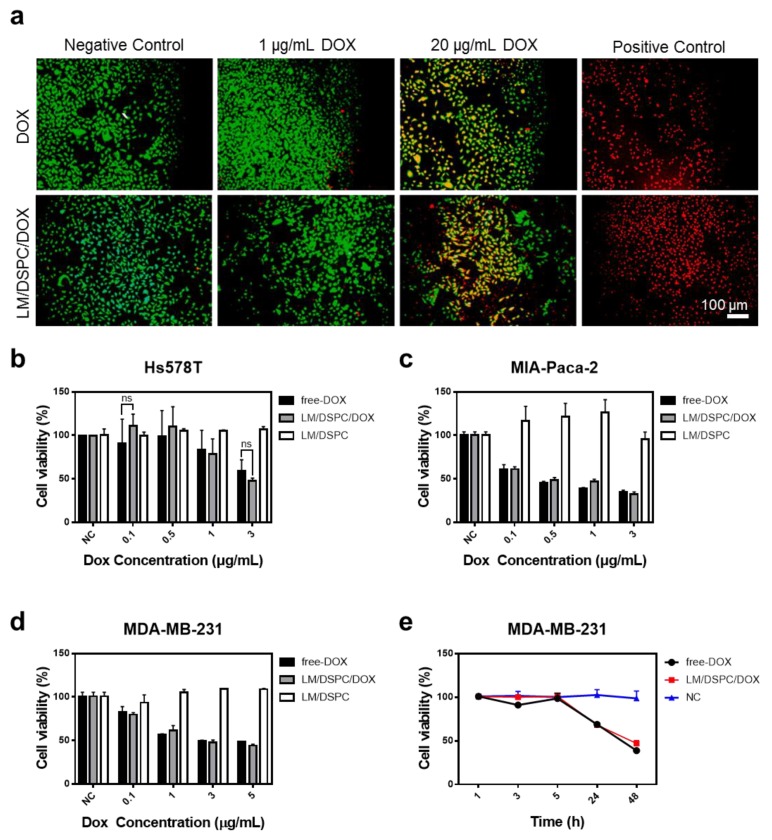
The effect of LM/DSPC/DOX particles on cell viability. (**a**) A live/dead cell viability assay showing live cells stained with calcein-AM (green) and dead cells stained with EthD-1 (red). (**b**) Viability of pancreatic cancer cells treated with LM/DSPC/DOX particles loaded with increasing concentrations of doxorubicin. (**c**,**d**) Viability of breast cancer cells treated with LM/DSPC/DOX particles loaded with increasing concentrations of doxorubicin. (**e**) The cytotoxicity of the nanoparticles on MDA-MB-231 cells depends on time (NC = negative control).

A live/dead assay was used on cancer cells to determine the drug delivery rate of the synthesized LM/DSPC/DOX particles and to determine any anti-cancer effects. MDA-MB-231 breast cancer cells were treated with nanoparticles containing the same concentration of doxorubicin as the control. After eight hours of incubation, the low (1 μg/mL) and high (20 μg/mL) concentrations of DOX-loaded experimental groups showed the same cancer cell killing capacity as the control group treated with free doxorubicin (Figure 4a, Supplementary Figure S4). This demonstrates that doxorubicin was effectively delivered to the cells by the LM/DSPC/DOX particles and that the LM/DSPC/DOX particles are more effective at causing cancer cell necrosis than free doxorubicin.

### 3.4. Cell Viability Test Using LM/DSPC and LM/DSPC/DOX Particles

As most metal particles are toxic, we performed cytotoxicity experiments in cells treated with LM/DSPC particles without DOX loading. LM/DSPC particles did not exhibit cytotoxicity in the three types of cancer cells evaluated (Figure 4b–e). Several types of cancer cells were treated with LM/DSPC/DOX particles using various concentrations of doxorubicin, and cell viability was analyzed. Particles added to Hs548T cancer cells showed statistically insignificant anti-cancer effects when compared with the cytotoxicity caused by free doxorubicin (Figure 4b). Treatment of MIA-Paca-2 cells and MDA-MB-231 cells showed similar results (Figure 4c,d).

MDA-MB-231 cancer cells were used to investigate the hourly drug delivery of LM/DSPC/DOX particles. The cell survival rate was similar to that of the control group. After 24 h of particle and drug treatment, 40% of the cells died, and after 48 h, 60% of the cells died (Figure 4e). These results indicate that LM/DPSC/DOX particles, per se, are not cytotoxic, are efficiently absorbed into cells, and have the same anticancer effects as free DOX in several types of cancer cells.

### 3.5. Shape Transition of LM/DSPC Particles Caused by Light and Heat Treatment

**Figure 5:**
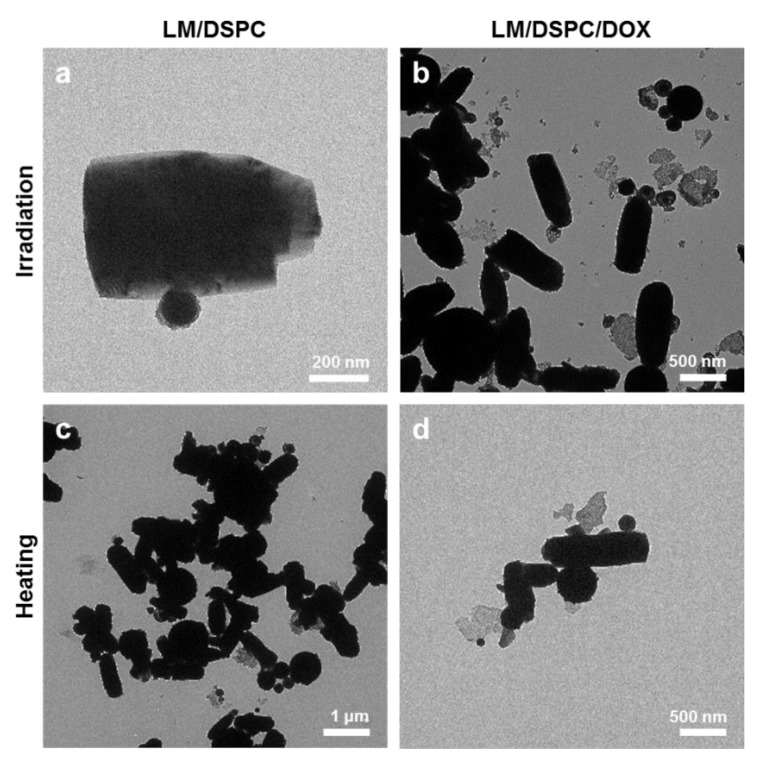
Morphological transformation of LM/DSPC and LM/DSPC/DOX particles. (**a**,**b**) TEM images of LM/DSPC and LM/DSPC/DOX particles after laser exposure. (**c**,**d**) TEM images of LM/DSPC and LM/DSPC/DOX particles after heating them to 70 °C.

The photothermal characteristics and temperature-dependent changes of the LM/DSPC and LM/DSPD/DOX particles were confirmed by TEM after laser irradiation and heat treatment (Figure 5, Supplementary Figure S5). Upon laser treatment, the shape of the two particles changes in a similar fashion, forming rods of around 500 nm in length (Figure 5a,b). The particles showed a similar tendency upon heat treatment, indicating that their change in shape is due to temperature (Figure 5c,d). In Supplementary Figure S5g–i, the shape change at 50, 60, and 70 °C is shown. However, because it takes a long time to reach a temperature that can cause shape deformation, it will be necessary to design particles that can change their shape more rapidly. In addition, when the size of the particles before and after the heat treatment was measured by DLS and compared, the average diameter of the particles before the heat treatment was 400 nm, but the average particle size after the heat treatment increased to 600 nm. Also, the overall size distribution shifted toward larger diameters (Supplementary Figure S6). Owing to these changes in the shape of LM/DSPC particles, they are a potential vascular embolic material.

### 3.6. Transforming the Shape of LM/DSPC Particles Causes Membrane Occlusion

To determine whether LM/DSPC particles can cause a vascular embolism, a membrane passage experiment quantified the perfusion of the particles before and after temperature treatment via absorbance with a spectrophotometer (Supplementary Figure S7). The absorbance decreased 49%, indicating that LM/DSPC particles could pass through the membrane. After applying heat, the LM/DSPC particles agglomerated and failed to cross the membrane because of changes in their size and shape. These data illustrate their potential for blood vessel embolization.

### 3.7. Mimicking Vascular Embolization

**Figure 6:**
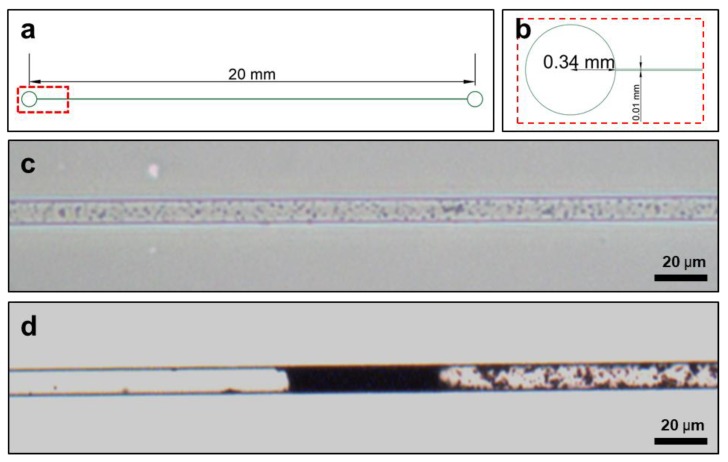
LM/DSPC/DOX particles tested on microfluidic chips as an embolism model. (**a**,**b**) The design of the PDMS microfluidic chip. (**c**) Fluid containing LM/DSPC/DOX particles in a micro channel. (**d**) Blocking the micro channel with heat induced transformation of LM/DSPC/DOX particles.

Experiments were performed to simulate vascular embolism by flowing LM/DSPC/DOX particles into a microfluidic chip (Figure 6). The particles were dispersed and flowed freely through the channel before heat treatment, but heat treatment caused the particles to change their shape, aggregate, and block the channel (Figure 6c,d). For a more detailed observation, the same experiment was conducted using 10 μL of LM/DSPC/DOX particles on a microfluidic chip with a depth of 50 μm and a width of 100 μm. At room temperature, the particles gathered via evaporation, but the particles did not block the channel. Upon heat treatment, however, the particles blocked the microchannel in the same way as the microfluidic chip with the smaller sized channels (Supplementary Figure S8). This verifies that LM/DSPC/DOX particles change their shape with heat and, consequentially, may be capable of inducing vascular embolism.

## 4. Conclusions

In this study, we developed a nanoparticle drug carrier and vascular embolic agent that can be applied to tumors that cannot be removed by surgery. The nanoparticles contained an EGaIn core and a lipid DSPC-containing surfactant (DSPE-PEG-2000) containing the anticancer drug doxorubicin. They absorb light at all wavelengths, similar to EGaIn, and have a diameter of approximately 500 nm. This allows them to be treated with infrared light, which is more suitable for in vivo applications. When cancer cells were treated with LM/DSPC/DOX particles, it was confirmed that the particles located to the cell periphery because of their lipid component, and the drug penetrated the cells and was transported to the nucleus. LM/DSPC/DOX particles showed similar anticancer effects to doxorubicin, alone, in several cancer cell types. In particular, the anti-cancer effects of free-dox and LM/DSPC/Dox were the same (approximately 50%) after 24 h of Dox (3 μg/mL) treatment. The potential of LM/DSPC/DOX particles as a vascular embolic agent was also demonstrated by their ability to block microfluidic channels after heat treatment. After 10 μL of particles (5 mg/mL) was injected and processed at 70 °C for 30 min, they successfully blocked the channel flow.

One major limitation of working with LM particles is that changing their shapes in a uniform fashion is difficult to achieve with a laser because it does not evenly transfer heat to the particles. In future experiments, it will be necessary to study methods that add polymers or nanoparticles to LM. Polymers and nanoparticles increase the efficiency of light/heat transfer, which causes LM particles to change their shape. In addition, the drug delivery properties of LM particles should be verified using 3D cell cultures to ensure the particles can efficiently change their shape at in vivo temperatures. LM particles should be tested in future animal experiments as a vascular embolizer for tumor tissues that cannot be treated surgically.

## Supplementary Materials

The following are available online at https://www.mdpi.com/2072-6694/11/11/1666/s1, Figure S1: Transmission electron microscopy (TEM) images of LM/DSPC, Figure S2: Field Emission Scanning Electron Microscope (FE-SEM) images of (a–c) LM/DSPC and (d–f) LM/DSPC/DOX, Figure S3: (a,b) Confocal microscopy of MDA-MB-231 breast cancer cell after incubation for 8 h with LM/DSPC and nuclei (blue). (c,d) Confocal laser scanning microscopy (CLSM) images of LM/DSPC/DOX and doxorubicin (red), Figure S4: Live/Dead cell viability assay. Cells were treated with (a) doxorubicin and (b) LM/DSPC/DOX, Figure S5: TEM images of morphological transformation of LM/DSPC after heating at (a–g) 70 °C, (h) 60 °C and (i) 50 °C, Figure S6: Dynamic light scattering (DLS) analysis. Size distribution of (a) LM/DSPC/DOX before heating and (b) after heating, Figure S7: Changes in absorbance of LM/DSPC, when LM/DSPC particles passed through the membrane. (a) UV spectroscopy of LM/DSPCs. (b) Membrane passed through the LM/DSPC particles before heating (c) after heating, Figure S8: Blocking of microfluidics channels with LM (channel width 100 μm) (a) Fluid of LM/DSPC/DOX micro/nano particles. (b) Dry the solvent at room temperature. (c) Induce the clogging of channel by heating LM/DSPC/DOX particles.
