# Supplementary material for: Effective Delivery of Anti-Cancer Drug Molecules with Shape Transforming Liquid Metal Particles
Source: Cancers (Basel). 2019 Oct 27;11(11):1666. doi: 10.3390/cancers11111666 (PMC6896188; doi:10.3390/cancers11111666)
Supplement: Supplementary file 1 [file cancers-11-01666-s001.pdf]

**Supplemental Materials**

# Effective Delivery of Anti-Cancer Drug Molecules with Shape Transforming Liquid Metal Particles

**Dasom Kim, Jangsun Hwang, Yonghyun Choi, Yejin Kwon, Jaehee Jang, Semi Yoon and Jonghoon Choi**

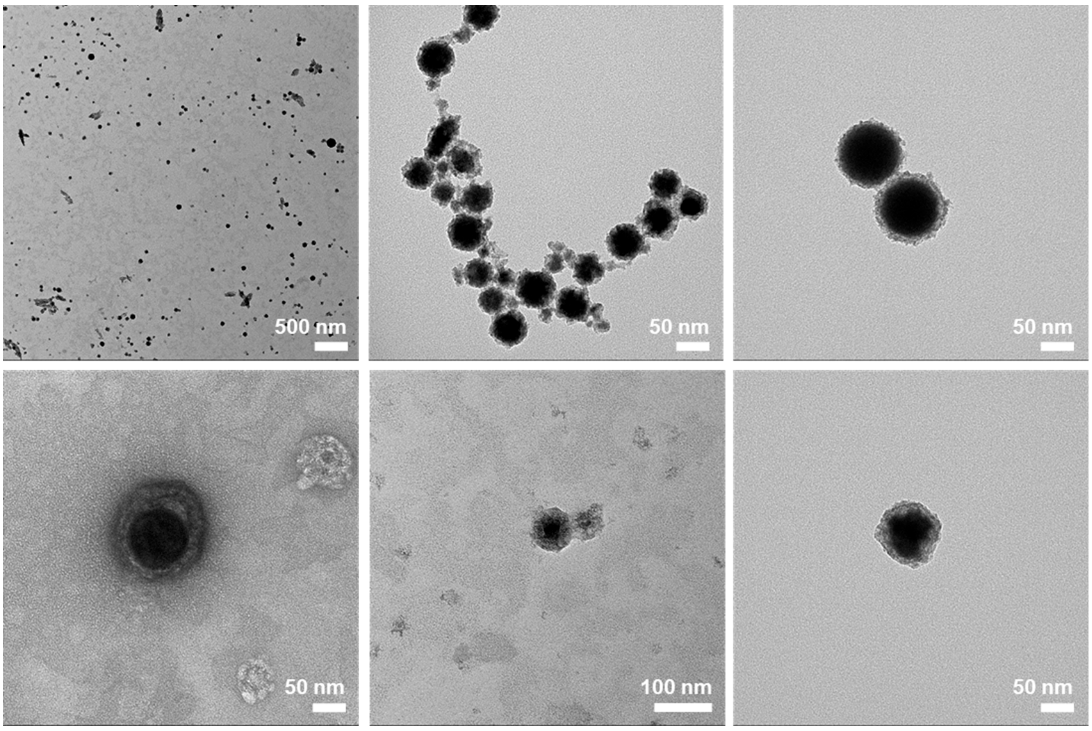


**Figure S1.** Transmission electron microscopy (TEM) images of LM/DSPC.

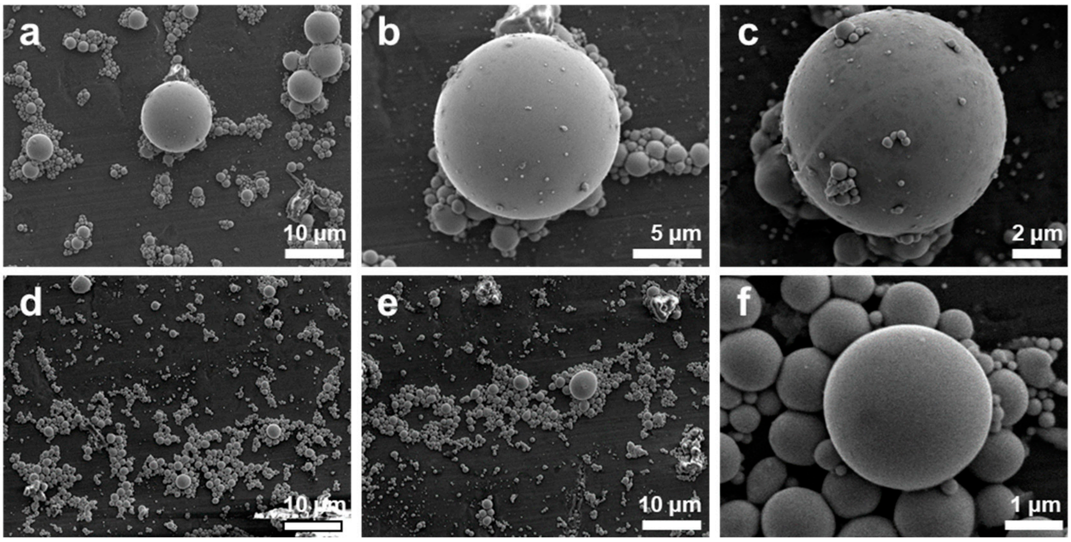


**Figure S2.** Field Emission Scanning Electron Microscope (FE-SEM) images of (**a**–**c**) LM/DSPC and (**d**–**f**) LM/DSPC/DOX.

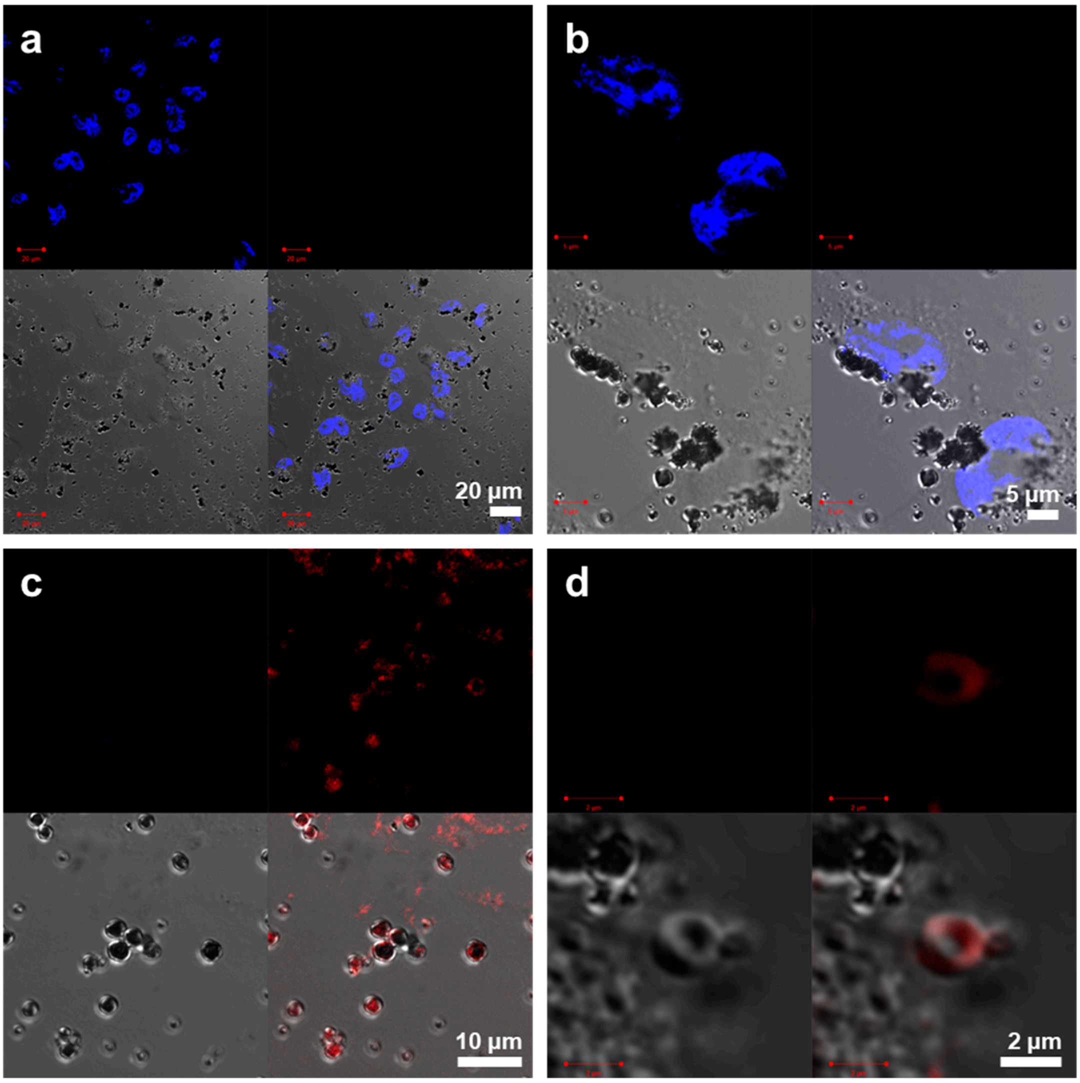


**Figure S3.** (**a**, **b**) Confocal microscopy of MDA-MB-231 breast cancer cell after incubation for 8 h with LM/DSPC and nuclei (blue). (**c**, **d**) Confocal laser scanning microscopy (CLSM) images of LM/DSPC/DOX and doxorubicin (red).

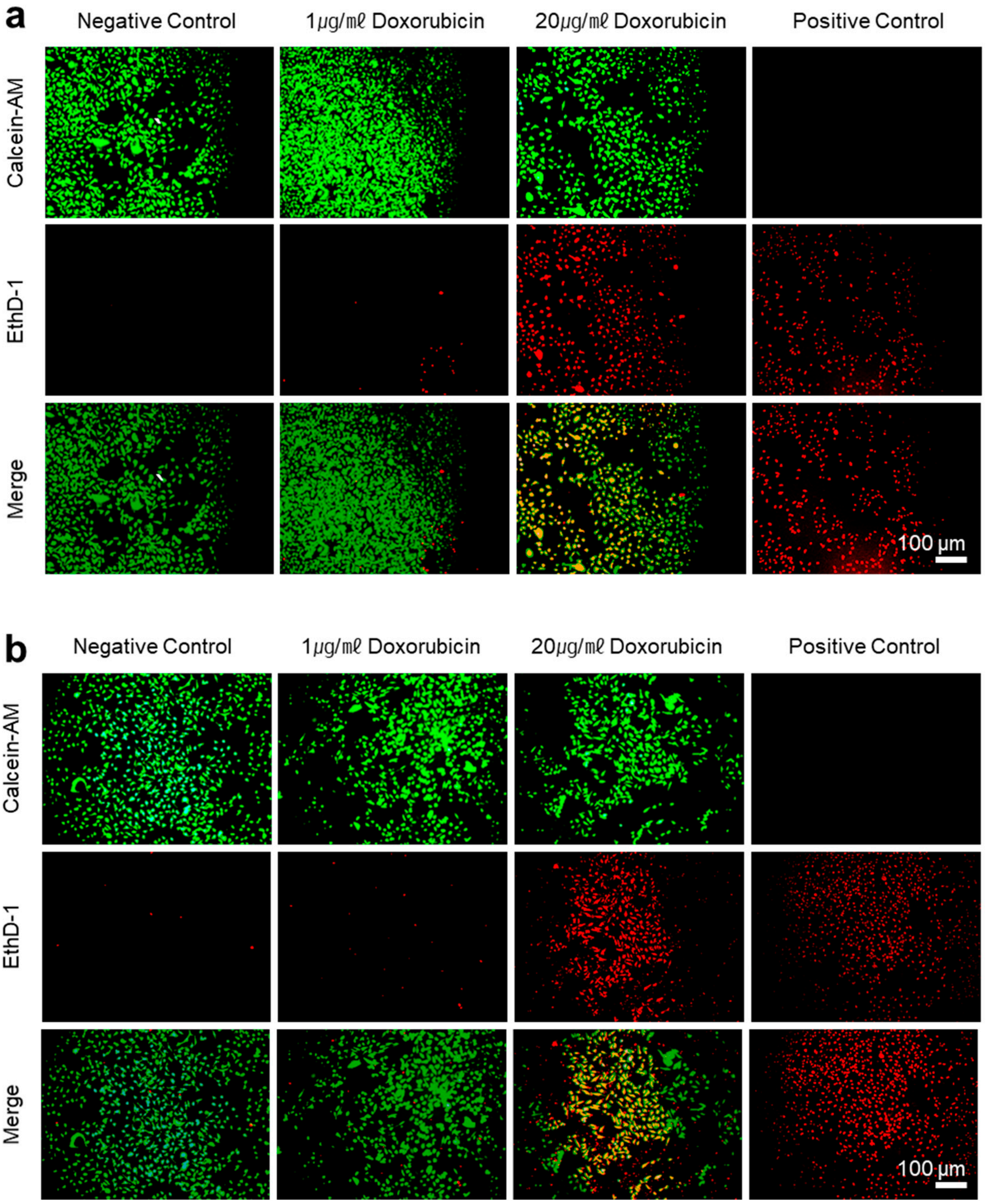


**Figure S4.** Live/Dead cell viability assay. Cells were treated with (**a**) doxorubicin and (**b**) LM/DSPC/DOX.

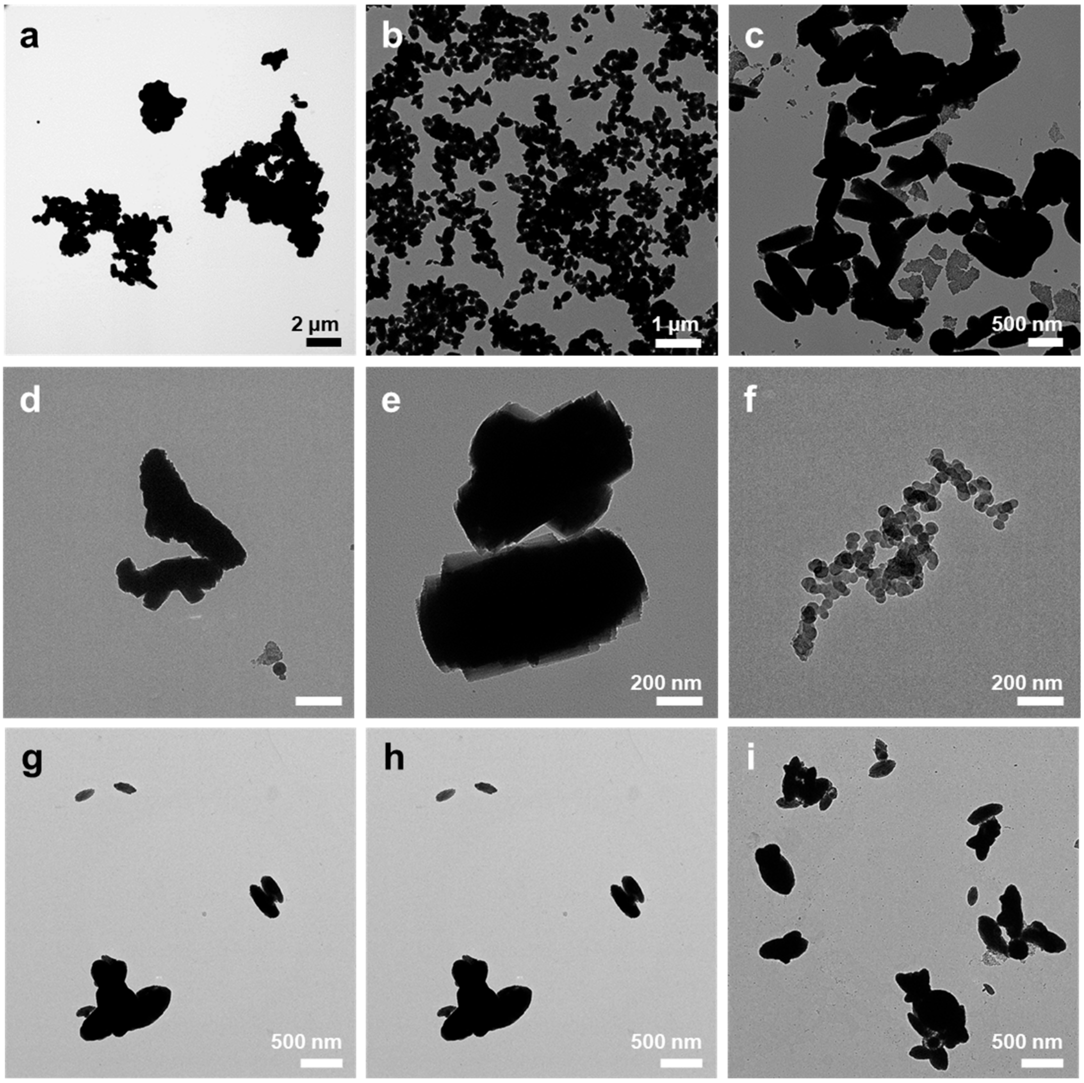


**Figure S5.** TEM images of morphological transformation of LM/DSPC after heating at (**a**–**g**) 70°C, (**h**) 60°C and (**i**) 50°C.

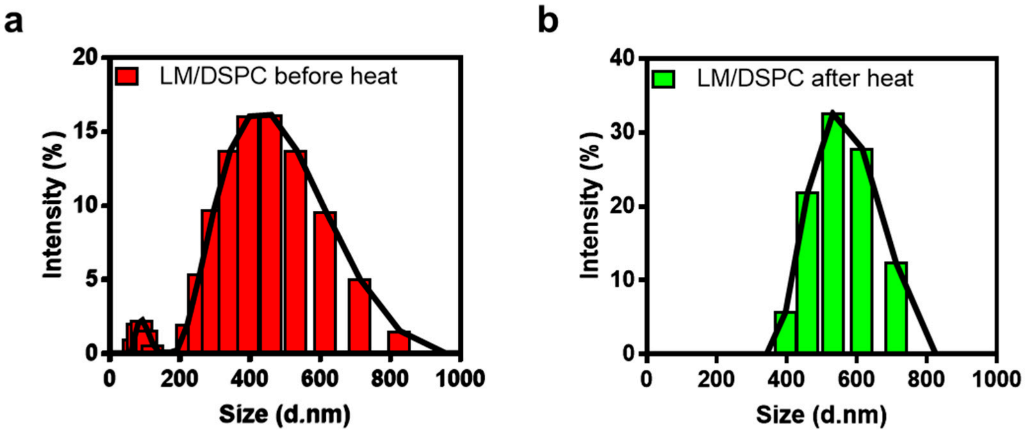


**Figure S6.** Dynamic light scattering (DLS) analysis. Size distribution of (**a**) LM/DSPC/DOX before heating and (**b**) after heating.

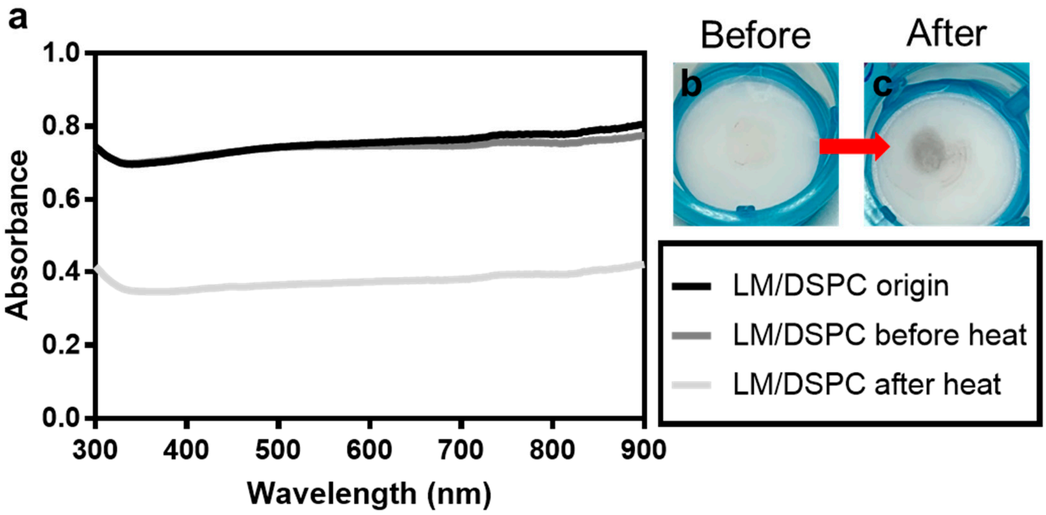


**Figure S7.** Changes in absorbance of LM/DSPC, when LM/DSPC particles passed through the membrane. (**a**) UV spectroscopy of LM/DSPCs. (**b**) Membrane passed through the LM/DSPC particles before heating (**c**) after heating.

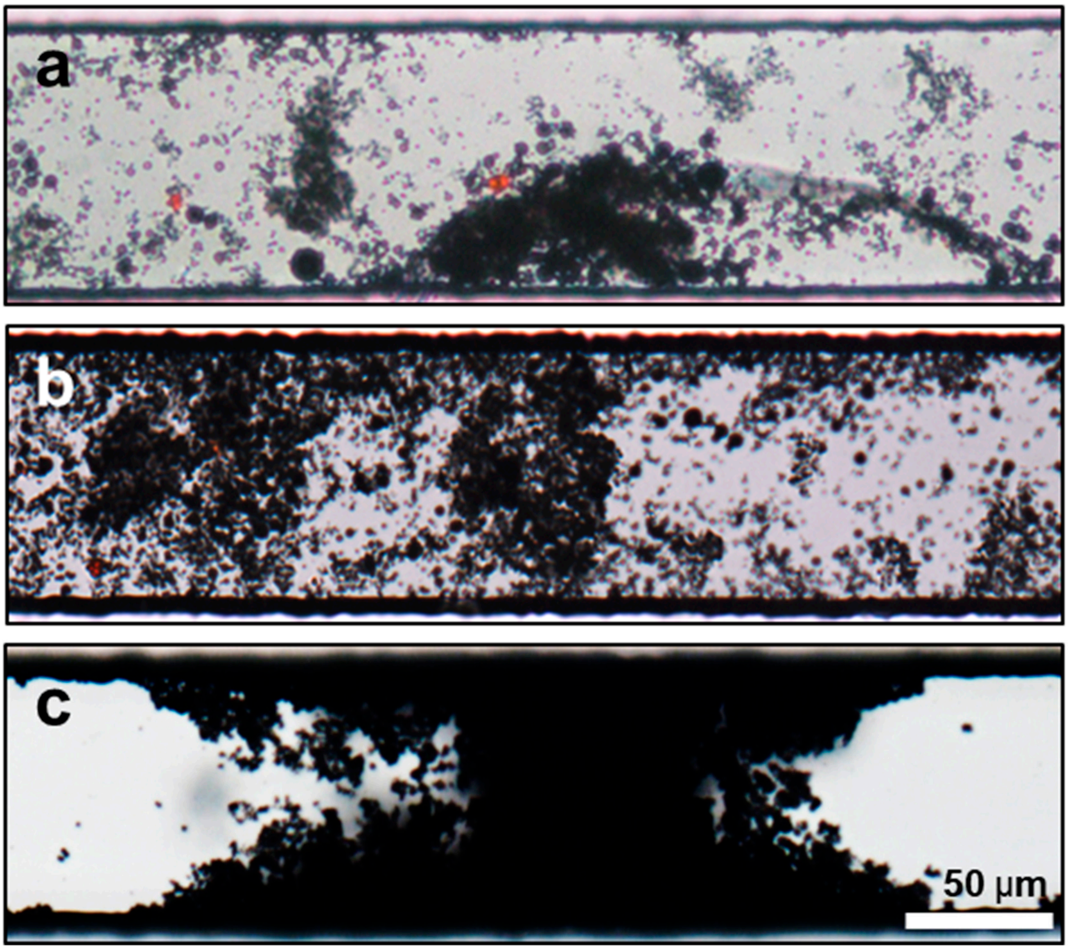


**Figure S8.** Blocking of microfluidics channels with LM (channel width 100 μm) (**a**) Fluid of LM/DSPC/DOX micro/nano particles. (**b**) Dry the solvent at room temperature. (c) Induce the clogging of channel by heating LM/DSPC/DOX particles.
